# Should Every Cow Carry a Government Health Warning?

**Published:** 1987-02

**Authors:** J. R. A. Mitchell

**Affiliations:** Foundation Professor of Medicine, Nottingham University Medical School, Nottingham, England


### "Should every cow carry a government health warning"?

**J. R. A. Mitchell**
Foundation Professor of Medicine, Nottingham University Medical School, Nottingham, England

There's always an easy solution to every human problem—neat, plausible and wrong (H. L. Mencken)

Whenever anyone says "it is generally agreed that ..." it invariably means that it is neither known or agreed. In any field where no progress appears to be occurring, it is always useful to look at the points on which there is indeed general agreement because they may well be wrong. Even a brief glance at medical history should give us a due sense of humility when we look at the "neat and plausible" solutions of the past that are now perceived to have been mere dogma. This should make us wonder which of our present beliefs will not only be discarded but be actively derided by the year 2000.

A cosy and plausible chain of beliefs about diet and coronary heart disease (CHD) has reached the "it is generally agreed" category and runs as follows:

—CHD is caused by atherosclerosis
—Atherosclerotic plaques are cholesterol deposits in artery walls
—Atherosclerosis can be produced in animals by lipid feeding
—A high serum cholesterol is a risk marker for CHD
—Dietary lipids determine serum cholesterol

If one accepts that all these statements are true and that they belong to the same logic-chain, then two further statements arise:

—Dietary lipids cause coronary heart disease

As we shall see, most of the statements set out here are either untrue or irrelevant.

*CHD is caused by atherosclerosis* While the presence of underlying coronary artery-wall disease provides a necessary infrastructure for CHD, myocardial infarction and sudden death are rapid events. There is abundant evidence that the critical process in transmural myocardial infarction (Mitchell, 1978a) and in sudden death is thrombosis, often in association with plaque disruption (Davies & Thomas, 1984).

*Atherosclerosis is synonymous with lipid deposition* When Virchow (1858) looked at clinically-relevant artery-wall plaques he thought that they represented chronic inflammation. Observations showed him that all the layers of the artery wall were involved. The presence of lymphocytes, plasma cells, giant cells, fibroblasts and calcium deposits would not have permitted him to lend his name to any description of atherosclerosis that regarded it merely as lipid deposition in the intima.

*Atherosclerosis can be imitated in animals* Vascular lesions produced by feeding fat reflect the erroneous concept of human disease which we have already discarded since they merely show intimal foam cell/modified smooth-muscle-cell lesions which resemble clinically-irrelevant fatty streaks rather than multi-layer, multi-process, stenosing and thrombosing human lesions (Mitchell & Schwartz, 1965).

*Serum cholesterol predicts the risk of CHD* Since the earliest days of cholesterol measurement, it has been clear that for groups of individuals, it is a risk predictor for coronary events. Within groups, the link betwen serum cholesterol concentration and coronary risk is not a strong one. It is perfectly possible to have a heart attack with normal serum lipids and not to have a heart attack with elevated lipids, so cholesterol cannot stand in the same relation to CHD as was demanded of the acid-fast bacillus by Koch when he put forward his postulates about the proof required of a supposed cause for disease.

Association does not imply causality, but cholesterol has been assumed to play a prime causal role because of the 'articles of faith' set out previously. Moreover, one can blame the victim's life-style and can set about tidying it up, whereas for other equally-powerful risk markers one has to blame his parents and his genes. For example, short stature and high blood pressure have both emerged from major studies (Logan et al. 1978; Mitchell, 1978b) as risk predictors and yet both of them owe almost everything to inheritance and very little to environment. Social class is an extremely powerful mortality risk-marker (in the UK, the death-rates for men and women aged 15-64 years in the unskilled social class V are 2.5 times higher than in the professional social class I; Black et al. 1982). These are, however, not social classes but educational classes; in the USA, Hinkle et al (1966) found that asking healthy people about the duration and type of their education was just as powerful a predictor of CHD risk as cholesterol, smoking and hypertension. On these findings it would be as logical to assert that compulsory college education for all would halve the death toll from CHD as to make the more conventional claim that cholesterol-reduction would be similarly beneficial.

*Serum cholesterol is determined by diet* Cholesterol, like urate, is an endogenous material which in a free-range society is genetically determined and is minimally related to diet (Neufeld & Goldbourt, 1983). It is true that dietary change will modify serum cholesterol and that between-population comparisons show that total fat intake and saturation-level of that fat are correlated with serum cholesterol (Keys, 1980), but if one makes within-population comparisons, the influence of diet on cholesterol levels is minimal. Cholesterol is a marker of who you are rather than what you do.

*The theoretical claim* The case which is being put to the public rests on this series of statements, out of which emerges a rallying cry that poor diet causes coronary disease, so that by dietary manipulations we could prevent the epidemic of CHD. Before we examine the only way to test these claims, we need to be aware of information which is being used to lend support and credibility to them.

During the last 10 years, the USA coronary mortality has fallen by 25% (Harper, 1983) and there is an unseemly rush to claim the credit for this (prudent eating, better blood-pressure control, anti-smoking campaigns, coronary care units, jogging and weight reduction). The problem is that all sections of the USA community have been equally benefited and at the same time (old and young, rich and poor, black and white, men and women). I remain unconvinced that a poor black elderly woman living in Washington is likely to have changed her life style or been offered the advances in blood-pressure control and coronary care to the same extent as a young, affluent white man from Scarsdale. Those who believe that they can 'explain' the decline in CHD in the USA by better health care would do well to ponder why, in a highly-developed, health conscious and disciplined country like Sweden, CHD incidence and mortality seem to be increasing (Alfredsson & Ahlbom, 1983).

### THE FACTS

To those who keep saying that "better eating prevents coronary disease" we can accept that anyone is entitled to his beliefs but that scientists should be expected to produce evidence. The only acceptable evidence is an adequately-conducted clinical trial in which the outcome of a group who were given dietary advice aimed at lowering their serum lipids can be compared with a group who were not. If coronary disease is very common and kills many of its victims, then a reduction in coronary disease should be reflected in a fall in total mortality. Valid trials which fulfil these criteria are few in number and can be divided into those which dealt only with lipids and those which aimed at changing multiple risk-factors.

### Lipid Orientated Dietary Studies

*The Los Angeles Veterans Administration Study (Dayton et al 1969)*

846 men aged 55–89 living in a VA centre were randomly allocated to receive a low-cholesterol, low-saturated-fat, high-polyunsaturated diet, or to continue on the ordinary North American diet. The experimental group reduced their serum cholesterol by 13% during the 8-year follow-up period; the total deaths were 177 in the control group and 174 in the cholesterol-lowered group although the total mortality concealed a suggestion of a reduction in CHD deaths and an increase of 12% in non-CVS deaths.

### Multiple Risk-factor Trials

*The North Karelia Project* (Puska et al, 1983) Because Finland had the highest CHD mortality in the world, a community-based multiple-risk factor reduction strategy was adopted in North Karelia while the adjacent province of Kuopio served as a non-intervention control. In North Karelia there was an aggregated reduction of the main risk factors by 17% but on their original trial design, the comparison between the designated test and control areas was non-significant. To get a difference which was significant, they went beyond their original design to show that men in Karelia fared better in respect of CHD than in the rest of the country, minus Karelia. Women were no different on any analysis. In respect of total mortality, no demonstrable benefit emerged for any group.

*The Oslo Study* (Hjermann et al 1981). From 16,202 men aged 40–49, 1232 healthy men with elevated lipid levels were randomised into a 5-year study in which the intervention men were asked to stop smoking and to reduce their lipids by dietary means. During the trial, mean tobacco consumption per man fell 45% more in the intervention than the control group while the fall in cholesterol was only 13% greater in the intervention group. Had significant differences in outcome emerged, it would thus have been difficult to disentangle the benefit of stopping smoking from any effect of lipid reduction. However, the trial results were not conclusive (total mortality; control – 38 per 1000; intervention – 26 per 1000 which was not significant; fatal and non-fatal infarction plus sudden death was 47% lower in the intervention group; P=0.03).

*The United States Multiple Risk Factor Intervention Trial – MR FIT 1982* took 12,886 high-risk men and randomly allocated half to a special intervention group (SI) who had a programme of advice aimed to reduce blood pressure, smoking and plasma lipids, to conquer obesity and to increase physical activity. The comparative group, who knew of course that they were "high risk" were simply sent back to their doctors for "usual care" (UC), but as in Finland, this 'control' group changed their behaviour markedly, so both groups showed a fall in plasma lipids. Table I shows the end-result, in that the "got-at" men did slightly worse in terms of overall mortality than the "laissez-faire" men.

*WHO European Collaborative Group Study (1983)* 49,781 men aged 40–59 working in 66 factories were recruited. The factories were paired and one of each pair was randomly allocated to receive special intervention. Within an intervention factory the intention was to lower cholesterol by diet, to reduce smoking and weight, to increase physical activity and to control high blood pressure.

Table II shows how the risk factors fared and Table III shows the effect on the pre-determined end-points. Special intervention is clearly not good news for Britons in that they fared worse in all these end-points than their fellows who were left alone. (Table IV)

### Non-dietary lipid trials

*The Lipid Research Clinics Coronary Primary Prevention Trial (1984)*

They screened 480,000 men and identified the 3806 with cholesterol levels in the top 5% of the distribution. These men were all given cholesterol-lowering diets and only those whose serum lipids did not fall to predetermined levels went on into the oral cholestyramine versus placebo phase of the study. Thus the trial results are based on diet-resistant patients who were then followed for a mean of 7.4 years. The total deaths in the cholestyramine-treated group (n=1906) were 68 and 71 in the placebo group (n=1900), so screening half-a-million men and subjecting nearly 2,000 of them to 'treatment' for 7 years has 'saved' 3 lives. The trial organisers clearly perceived the unacceptability of a drug-based approach so wrote "the LRC-CPPT results and those of similar trials thus suggest that the risk of an initial CHD episode in hypercholesterolaemic middle-aged men can be reduced by half with currently available appropriate cholesterol-lowering agents and diets" even though their trial offers no evidence on the value of diet.

**Table 1**

**Results of MR FIT after 7 years**

| | SI | UC |
|---|---|---|
| Total mortality/1000 | 41.2 | 40.4 |
| CHD mortality/1000 | 17.9 | 19.3 |

**Table 2**

**Effect on risk factors (%) in WHO factory study**

| | UK | Belgium | Italy |
|---|---|---|---|
| Cholesterol | −0.4 | −0.9 | −4.8 |
| Cigarettes/day | −15.6 | −3.7 | −5.5 |
| Weight | −0.4 | +0.2 | −1.9 |
| Systolic BP | −1.6 | −2.3 | −4.1 |

**Table 3**

**WHO Factories Study (1986)**

| Group | n | Fatal CHD | | Non-fatal MI | | Total deaths | |
|---|---|---|---|---|---|---|---|
| | | n | % | n | % | n | % |
| Control | 26,971 | 398 | 1.5 | 505 | 2.1 | 1186 | 4.4 |
| Intervention | 30,489 | 428 | 1.4 | 505 | 1.9 | 1325 | 4.3 |
| Reduction % | | −6.9 | | −14.8 | | −5.3 | |
| Confidence limits | | −19 to +7 | | −28 to +1 | | — | |
| p | | 0.8 | | 0.06 | | 0.4 | |

**Table 4**

**Net % difference in outcome between groups in WHO factory study**

| | UK | Belgium | Italy |
|---|---|---|---|
| Fatal CHD | +8 | −21 | −30 |
| Total CHD | +5 | −24 | −14 |
| Total mortality | +14 | −17 | −6 |

### WHAT DOES IT ALL MEAN

What interests patients is staying alive and free from disability. They are not interested in risk-markers such as blood-lipids and blood pressure but only in their effect and in the benefit which modification of these factors will confer on them.

Once we have told our patients to stop smoking, then as scientists and men of commonsense, we have a duty to share our uncertainties about the other risk markers with them. If we do not do so, and they challenge us to produce evidence that by following the advice given by the Multiple Risk Factor Intervention Trial Research Group (1984) about diet, weight, activity and blood press-laborative Group, 1983 and the Lipid Research Clinic Group 1984) about diet, weight, activity and blood pressure control they will live longer or stay free from clinical CHD, then we cannot do so. The campaigners for mass prevention then turn on to another tack by saying: 'Even if it does no good it can do no harm'. I do not think that they have taken a broad enough view of what constitutes harm for there will be harm to our credibility as scientists and as health advisers. If we go public on diet and CHD on the basis of poor evidence we are weakening our position in respect of measures for which there is clear evidence: that everyone should be a non-smoker and that blood pressure control prevents strokes and heart failure. Over the centuries the public have been given so many crazy admonitions by "leaders" of medical opinion (on the evils of masturbation and of constipation; on the best way to treat mental illness, multiple sclerosis and breast cancer) that they cannot decide who to listen to and what they should do. If we fail in our duty to differentiate between what is known and what is merely believed, then the harm from such false advice is to our credibility, for as Mencken reminds us, "The most costly of all follies is to believe passionately in the palpably-not-true"

### REFERENCES

ALFREDSSON, L,. & AHLBOM, A. (1983) British Medical Journal **286,** 1931–1933.

BLACK, D., MORRIS, J. N., SMITH, C. & TOWNSEND, P. (1982) Inequalities in Health, p 57 Harmondsworth: Penguin Books.

DAVIES, M. J. & THOMAS, A. (1984) New England Journal of Medicine **310,** 1137–1140.

DAYTON, S., PEARCE, M. L., HASHIMOTO, S., DIXON, W. J. & TOMIYASU, U. (1969) Circulation **40,** Suppl. 2 1–63.

HARPER, A. L. (1983) American Journal of Clinical Nutrition **37,** 669–681.

HINKLE, L. E., BENJAMIN, B., CHRISTENSON, W. N. & ULLMANN, D. S. (1966) Archives of Environmental Health **13,** 312–321.

HJERMANN, I., HOLME, I., BYRE, K. V. & LEREN, P. (1981) Lancet **ii,** 1303–1310.

KEYS, A. (1980) Seven Countries. Cambridge, Mass: Harvard University Press.

LIPID RESEARCH CLINICS PROGRAM (1984) Journal of the American Medical Association **251,** 351–74.

LOGAN, R. L., RIEMERSMA, R. A. & THOMPSON, M. A. (1978) Lancet **i,** 949–955.

MENCKEN, H. L. In Peter L. Quotations for our time. Magnum London 1980 p 41.

MITCHELL, J. R. A. (1978a) British Medical Bulletin **34,** 103–106.

MITCHELL, J. R. A. (1978b) British Medical Journal **i,** 127–128.

MITCHELL, J. R. A. & SCHWARTZ, C. J. (1965) Arterial Disease. Oxford: Blackwell Scientific.

MULTIPLE RISK FACTOR INTERVENTION TRIAL RESEARCH GROUP (1982) Journal of the American Medical Association **248,** 1465–1477.

NEUFELD, H. N. & GOLDBOURT, U. (1983) Circulation **67** 943–954.

PUSKA, P., SALONEN, J. T., TUOMILEHTO, J., NISSINEN, A. & KOSKELA, K. (1983) Lancet **ii,** 406–407.

VIRCHOW, R. (1858) Die Cellular Pathologie. Berlin: Hirschwald.

WHO EUROPEAN COLLABORATIVE GROUP (1983) European Heart Journal **4** 141–147.

